# Integrated Pose Estimation Using 2D Lidar and INS Based on Hybrid Scan Matching

**DOI:** 10.3390/s21165670

**Published:** 2021-08-23

**Authors:** Gwangsoo Park, Byungjin Lee, Sangkyung Sung

**Affiliations:** Department of Aerospace Information Engineering, Konkuk University, Seoul 05029, Korea; pks87@konkuk.ac.kr (G.P.); schumir@konkuk.ac.kr (B.L.)

**Keywords:** scan matching, registration, normal distribution transform, localization, pose estimation

## Abstract

Point cloud data is essential measurement information that has facilitated an extended functionality horizon for urban mobility. While 3D lidar and image-depth sensors are superior in implementing mapping and localization, sense and avoidance, and cognitive exploration in an unknown area, applying 2D lidar is inevitable for systems with limited resources of weight and computational power, for instance, in an aerial mobility system. In this paper, we propose a new pose estimation scheme that reflects the characteristics of extracted feature point information from 2D lidar on the NDT framework for exploiting an improved point cloud registration. In the case of the 2D lidar point cloud, vertices and corners can be viewed as representative feature points. Based on this feature point information, a point-to-point relationship is functionalized and reflected on a voxelized map matching process to deploy more efficient and promising matching performance. In order to present the navigation performance of the mobile object to which the proposed algorithm is applied, the matching result is combined with the inertial navigation through an integration filter. Then, the proposed algorithm was verified through a simulation study using a high-fidelity flight simulator and an indoor experiment. For performance validation, both results were compared and analyzed with the previous techniques. In conclusion, it was demonstrated that improved accuracy and computational efficiency could be achieved through the proposed algorithms.

## 1. Introduction

For the past ten years, navigation research for autonomous vehicles such as drones, cars, and mobile robots has been extensively exploited in association with onboard sensor implementation diversity. The Global Positioning System (GPS) has been the most representative and widely used system, suitable for open-sky environments such as outdoor and rural areas [1]. However, as the vehicle gradually enters urban and indoor areas, reliability degradation mostly occurs caused by satellite signal denial and multipath errors. To overcome this, various studies investigating autonomous operation [2,3,4,5,6] have emerged considering these environments, as famously represented by SLAM (Simultaneous Localization and Mapping) [4,5,6].

SLAM consists largely of components that generate a map of the surrounding environment and estimates location. It can also be divided into visual SLAM, Lidar-based SLAM, or a hybrid of Lidar and visual SLAM, depending on the primary sensor implementation [6]. Traditionally, cameras have been most widely used, yet recent works employing lidar have increased outstandingly, owing to the sensor’s effective dissemination. In practice, the lidar mounted on the vehicle measures the distance and intensity and thus provides point cloud data against surrounding areas. Then the accumulated measurement is processed to generate a 3D map and to estimate the pose of an onboard vehicle through a registration procedure with the constructed map.

The point cloud registration algorithm (sometimes called scan matching) has been an important topic in computer vision (i.e., image processing) and robotics, which finds the best transformation (e.g., rotation and translation) that matches two different point cloud sets. Traditionally, the Iterative Closest Point (ICP) algorithm [7], which allows the distance between two data set points to be minimal through a repetitive performance inspection, has been widely used, and various improvements are still made, such as NICP [8] and voxelized GICP [9]. In general, ICP results do not always guarantee global optimal performances and are highly dependent on its initial guess. Given an incorrect initial pose, the ICP-based method can generate a local optimal or wrong solution. In addition, there is a disadvantage in that the amount of computation increases in proportion to the amount of point cloud data. This feature leads to disseminating the Normal Distribution Transform (NDT) algorithm [10,11]. NDT, unlike ICP, is a method employing the Gaussian probability distribution for scan matching, where point cloud map is voxelized for a point-to-point matching process. As a result, the impact of increasing point cloud data can be minimized, thus the NDT has been demonstrated to be more robust and accurate in real-time operation than ICP [12]. Due to these advantages, NDT has been widely adopted in autonomous vehicles [13,14].

Besides this, various kinds of matching algorithms [15,16,17] have been reported according to each performance requirement in the applications. Polar Scan Matching (PSM) [15] is a method belonging to a point-to-point matching category, which has faster characteristics than ICP because burdensome point-to-point search processes can be avoided by simply matching the association between points with the same bearing angle. In order to take advantage of the laser scanner measurement structure that outputs the distance to the bearing, the lidar polar coordinate system is mainly used. The proposed algorithm has been validated with kalman filter SLAM using a SICK LMS 200 laser range finder. Correlative Scan Matching (CSM) [16] is a scan matching algorithm based on cross-correlation between point cloud data. In obtaining the posterior distribution probability for the robot’s pose, Bayes’ rule and the pre-built 2D lookup table are used for accelerating computation. In [16], experimental results report robustness to initial noise and outperformance of existing ICP and ICL. Coherent Point Drift (CPD) [17] is an approach to find a solution by rephrasing the point set matching problem into a formulation of probability density estimation. To achieve this, it is used to align the measured dataset in the direction where the likelihood is maximized around the center of the Gaussian Mixture Model (GMM). The algorithm is characterized by reducing computational complexity through fast gauss transform and low-rank matrix approximation.

In each application, the algorithm varies according to its sensor configuration, measurement type and the corresponding performance requirements. In this study, a unique measurement environment containing edges and vertices, such as transmission tower and truss bridge, is considered for its application. We assume a 2D lidar measurement in these conditions, while a 3D map is available for point cloud registration. Especially, a new scan matching technique is developed such that the probability distribution of scan points and the score function are uniquely constructed for best resolution of the pose estimation problem. Compared with the typical NDT algorithm, the proposed method suggests that feature points (e.g., corner) are distinctively extracted from the point cloud data, then the accumulated feature points approximate the covariance of the distribution by which the score function of the distribution is effectively updated.

As noted, this paper mainly focuses on developing a localization technique using lightweight point cloud data from 2D lidar while the map is already implemented. In this context, computational efficiency with comparable estimation performance can be regarded as essential design criteria. Thus, quantitative analysis with the existing scan matching methods is included for performance comparison, where both simulation and experimental results are employed to demonstrate each algorithm’s performance. This paper is organized as follows: Section 2 presents related work on the registration algorithm based on NDT. In Section 3, we describe in detail the NDT-based algorithm with feature points. Next, we conduct the simulation and experimental to validate and show the results. Finally, the paper concludes and suggests future work.

## 2. Related Work

In this section, a brief literature survey of the related topic is summarized. First, as a basis of the proposed algorithm, NDT was introduced by Bieber et al. in 2003 [10] and expanded in three dimensions by Martin Magnusson in 2006 [11]. NDT is a method for matching scan points to target points through the underlying probability condition in which the scan point measurements from lidar serve as input, and the prebuilt 3D point cloud map provides target points. Typically, the target points in the map are preliminarily voxelized, then the map is divided into cells of a uniform size for calculating the mean and covariance of each cell. Next, each scan point is corresponded with cells to solve an optimized target point. Voxelization can improve computational power by reducing the number of target points by the size of the voxel grid. In principle, the original method can be seen as a point-to-distribution (P2D) approach as it directly matches the scan points and the probability distribution of the voxelized targets. Afterward, Stoyanov applied probability methods for both scan points and target points to improve registration speed [18].

Map accuracy also influences the performance of the registration algorithms. In 2013, Sarrinen [19] represented a new 3D space called Normal Distribution Transform Occupancy Map (NDT-OM) that combines the advantages of occupancy grid maps with NDT maps. The occupancy grid map has such a robustness that only stationary parts of the surrounding environment are generated in a representational form based on the probability of cell occupancy. On the other hand, the NDT map has the advantage of compactness by voxelizing the point cloud map to express each cell in a gaussian probability density. Still, the disadvantage is that traces remain together in a dynamic environment where new objects can frequently appear and disappear. By dividing the space into a uniform grid size, the geometric features of the point’s continuity and local space may also disappear. To solve these problems, Composite Clustering NDT (CCNDT), which calculates the probability distribution with the clustered points, was introduced in 2020 by Liu et al. [20]. This work suggested the application of a probability distribution using Density-Based Spatial Clustering with Applications (DBSCAN) and k-mean clustering methods, rather than a grid of constant size. Consequently, the presented method could maintain both local features while maintaining the continuity of target points.

In 2017, Andreasson et al. [21] extended the cost constraint by adding full pose information to the NDT-D2D (distribution-to-distribution) [18] approach. Previously, ego-motion estimation (e.g., odometry) was simply used as an initial pose guess. However, in the presented work, it is suggested that the objective function incorporates ego-motion estimation with uncertainty, thus its performance is revealed to be more accurate under feature-poor and self-similar environments. In 2018, Liu et al. [22] proposed an Improved NDT (INDT) that registers using fractional pre-processed feature points only. For this, the work applied a Fast Point Feature Histogram (FPFH) descriptor and Hausdorff distance method to extract feature points. The presented method also contributed to improving accuracy by replacing single-Probability Density Functions (PDF) with mixed PDFs. The papers of [23,24] introduced an NDT-ICP algorithm that combines and operates NDT and ICP sequentially. The suggested method divides the registration process into two stages and performs a coarse registration with NDT. Then, a fine registration is carried out with the ICP process. Through this hybrid mechanism, the coarse registration result of NDT could be significantly enhanced.

In relation to the previous literature, the features of this paper are characterized as follows. Assuming a pre-generated map, a voxelized NDT map with constant grids commonly used in SLAM is employed. The adapted score function of the proposed algorithm has similarities with the previous reference [21], yet the objective function employs a unique classifier coefficient depending on point characteristics. The INDT approach in [22] also extracts and uses feature points during the scan matching procedure, yet differs from the proposed methods in that extracted feature points are only used for the registration process. Due to this reasoning, the INDT in [22] is excluded from the performance comparison algorithm because poor performance is achieved through INDT with a relatively smaller number of feature points for the 2D measurement environment. On the other hand, the NDT-ICP method in [23], which performs NDT and ICP sequentially, is implemented as a performance comparison algorithm. In view of hybrid implementation, the proposed approach has similarity, but it has the advantage of executing NDT and ICP in parallel during each registration period for computational efficiency. On the other hand, the proposed algorithm has limitations as follows. First, 2D lidar measurements are fundamentally assumed, which can be vulnerable to altitude estimation. If there are few candidates for feature extraction, the proposed method simply degenerates into a NDT scheme without effective performance enhancement.

The proposed algorithm details are described in Section 3. The main contributions of the study are summarized as follows.

(1) Develops classification and integration mechanism with different point clouds, allowing both point-to-point and point-to-distribution based scan matching.
(2) Adapts the uncertainty of feature points into the pose optimization scheme.
(3) Presents localization accuracy in the real world through INS integration.
(4) Validates the proposed algorithm through the results of the reference method from both simulation and experiments.

## 3. Algorithm Implementation

In this section, the fundamentals of a registration algorithm are illustrated in association with the NDT. Then a modified registration concept is put forward that integrates point-to-point matching with distribution-to-point matching framework.

### 3.1. NDT Formulation

NDT is a representative registration algorithm that matches a two-point cloud data set based on probability information. For this, the map is voxelized such that it divides the map into a cell of a uniform size. The mean μ and covariance Σ of the included points $m_{i}\;\left(i=1,\cdots ,n\right)$ in each cell are computed as

(1) $$\mu =\frac{1}{n}\sum_{i=1}^{n}m_{i}$$

(2) $$\Sigma =\frac{1}{n}\sum_{i=1}^{n}\left(m_{i}-\mu \right){\left(m_{i}-\mu \right)}^{T}$$

**Figure 1:**
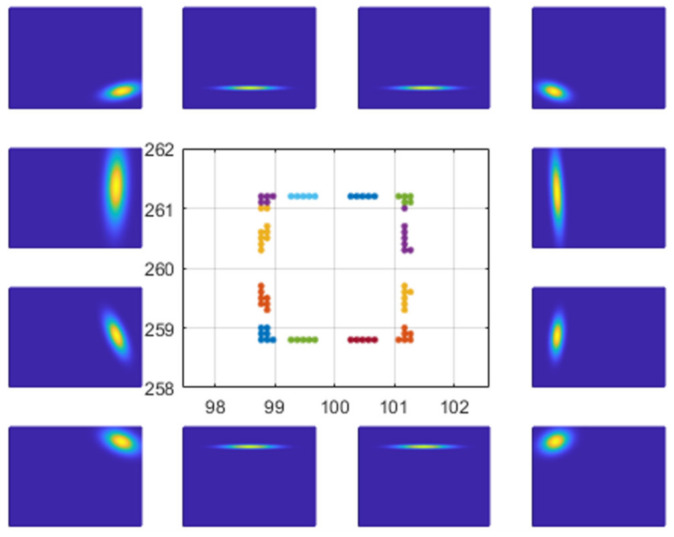
NDT Map.

Then a normal distribution $N\left(\mu ,\Sigma \right)$ for scan point $x_{i}\;\left(i=1,\cdots ,n\right)$ measured by lidar is generated, using the previously obtained mean and covariance. The Probability Density Function (PDF) for $x_{i}$ is described as

(3) $$p\left(x_{i}\right)=\frac{1}{\sqrt{{\left(2\pi \right)}^{D}\left|\Sigma \right|}}\exp\left(\frac{-{\left(x_{i}-\mu \right)}^{T}\Sigma^{-1}\left(x_{i}-\mu \right)}{2}\right)$$

where D is a dimension of scan point $x_{i}$, and PDF $p\left(x_{i}\right)$ is the probability that scan points $x_{i}$ will be included in cells with a normal distribution $N\left(\mu ,\Sigma \right)$. Figure 1 shows a visualization of the PDFs computed within each cell with 1m side length. This is called the NDT map. The higher the probability in each cell, the brighter and denser are the parts observed.

Given PDFs for scan points $x_{i}$, decision criterion on alignment is determined via the maximum sum of PDF. This sum is evaluated as a score of the transform parameter y, which can also be called the NDT score function, $s\left(y\right)$.

(4) $$s\left(y\right)=\sum_{i=1}^{n}p\left(T\left(y,x_{i}\right)\right)$$

(5) $$T\left(y,x_{i}\right)=R_{rot}\;x_{i}+t$$

where $y={\left[t_{x},t_{y},t_{z},\phi_{x},\phi_{y},\phi_{z}\right]}^{T}$ is the transform parameter, which includes translation $t={\left[t_{x},t_{y},t_{z}\right]}^{T}$ and rotation $\phi_{NDT}={\left[\phi_{x},\phi_{y},\phi_{z}\right]}^{T}$ about the x–y–z axis between two different point cloud data, and $T\left(y,x_{i}\right)$ is the transformation function, which transforms points $x_{i}$ by transform parameter y. This can be calculated as Equation (5). $R_{rot},t$ in (5) denotes the rotation matrix and translation vector, respectively. The next step is to optimize the score function in (5). The optimization problem is typically solved via numerical minimization framework. In this work, Newton’s method for finding the minimum value of the nonlinear function is selected. By setting the negative score function $-s\left(y\right)$, Newton’s method is described as

(6) H Δy=−g

(7) $$g_{i}=\frac{\partial s}{\partial y_{i}},\;\;\;\;H_{ij}=\frac{\partial^{2}s}{\partial y_{i}\;\partial y_{j}}$$

where H is the hessian matrix and g is the gradient of the score function. The solution Δy continues to be added in the current estimate until reaching convergence, and finally estimates the best y within tolerance for scan points to match the map.

(8) y←y+Δy

### 3.2. NDT-P2P

**Figure 2:**
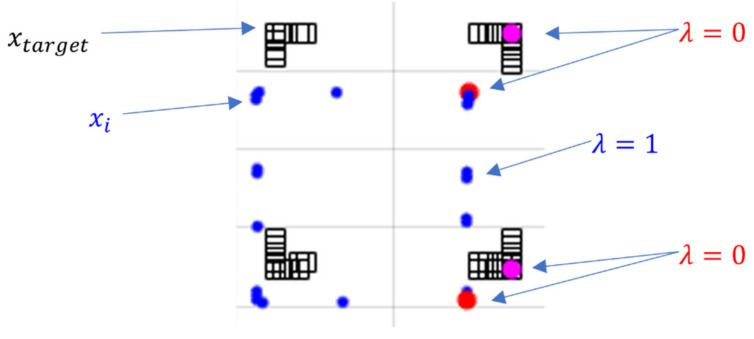
Point cloud map and point cloud data measured by 2D lidar.

In a typical NDT algorithm, all scan points are uniform in constructing normal distribution. Considering a navigation environment with frequent edges and vertices, we propose that scan points are divided into feature points and other points through a feature extraction procedure. Figure 2 shows an example of feature extraction around corners. Blue dot marker shows scan points measured by 2D lidar, and black square marker shows the point cloud map. Red & magenta dot markers represents corners. As shown in this case, scan points are divided into two categories according to their geometric distribution, where corners can be explicitly extracted as feature points. For extracting the feature point, the method in reference [25] is used.

In this study, a penalty coefficient λ is introduced to separately establish score functions during the scan optimization process. Specifically, penalty coefficient λ is set to λ=1 for the point cloud except for corner points. In this case, a normal NDT process for the corresponding points is taken, for which the probability distribution using the mean and covariance of the voxelized map is employed. In case of feature points such as corners, penalty coefficient is set to λ=0, thus a point-to-point matching process is applied. The accuracy of matching between points can be represented by the Euclidean distance with the following definition.

(9) $$d\left(x_{key},x_{target}\right)=∥x_{key}-x_{target}{∥}_{L2}$$

The subscript ‘key’, ‘target’ in (9) implies a corner of the scan points and a map point closest to a corner point. At this time, there is a data association problem in determining the map point $x_{target}$ corresponding to the corner point $x_{key}$. In this study, the shortest distance between points is obtained by using the *k*-Nearest Neighbor (KNN) algorithm. On the other hand, if feature points can be directly extracted on the map, these points serve as target points. In (10)–(11), probability density function and score function for the separated feature points are described.

(10) $$p\left(x_{key}\right)=\exp\left[-\frac{1}{2}{\left(x_{key}-x_{target}\right)}^{T}\Sigma_{key}^{-1}\left(x_{key}-x_{target}\right)\;\right]$$

(11) $$s\left(\vec{p}\right)=\sum \exp\left[-\frac{1}{2}{\left(x_{key}^{\prime }-x_{target}\right)}^{T}\Sigma_{key}^{-1}\left(x_{key}^{\prime }-x_{target}\right)\;\right]$$

where $\Sigma_{key}^{-1}$ is the inverse matrix of covariance for feature point, and $x_{key}^{\prime }$ is a transformed $x_{key}$ by y as a result of Equation (5). The probability depends on the distance between $x_{key}$ and $x_{target}$, and if $p\left(x_{key}\right)$ equals 1, then two points are considered to be matched.

**Figure 3:**
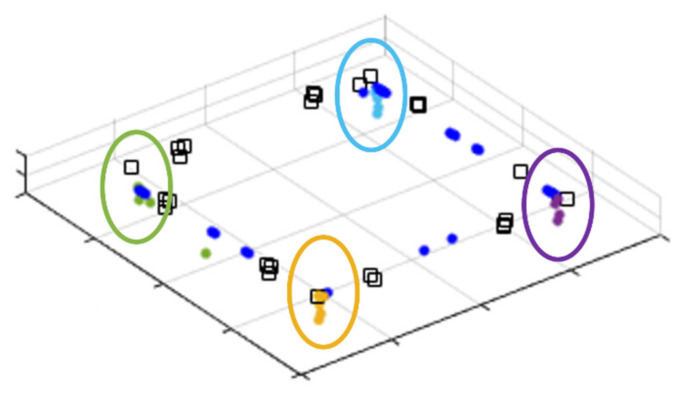
Accumulated corner extracted from scan points.

Uncertainty was obtained through the accumulation of corner points and applied to covariance $\Sigma_{key}$. The accumulated lidar measurements during the stationary period give noise characteristics for the point cloud data. If the points of a particular position measured by moving lidar are accumulated, the points will continue to be stamped in the same position with no errors in the ideal environment. With this idea, we stack the extracted feature points and obtain uncertainty from the accumulated points. Figure 3 illustrates the stacked corner points. The corners can be clustered into four sets. We obtained covariance from points within the window size using a moving window, and this value is applied to the point-to-point matching process.

**Figure 4:**
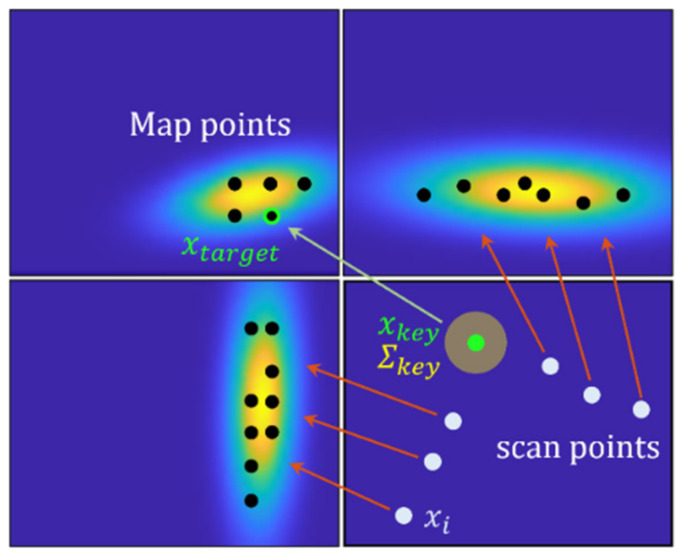
Correspondence between scan points and map points.

A hybrid formulation combining conventional NDT and pointwise matching for corner points is summarized in (12). The resulting correspondences between scan points and map points are illustrated in Figure 4.

(12) $$p\left(x\right)=\lambda \exp\left[-\frac{1}{2}{\left(x-\mu \right)}^{T}\Sigma^{-1}\left(x-\mu \right)\right]+\left(1-\lambda \right)\exp\left[-\frac{1}{2}{\left(x-x_{target}\right)}^{T}\Sigma_{key}^{-1}\left(x-x_{target}\right)\right]$$

(13) $$s\left(y\right)=-\sum p\left(T\left(y,x\right)\right)$$

where λ is the feature points delimiter among scan points, and λ equals 0 in the case of a point being a feature point; otherwise, λ equals 1. For example, if no feature points from scan points exist, this is the same as a normal NDT since λ is assigned to 1 for all points. Finally, the process of obtaining an optimal solution for a score function in (13) is numerically performed, taking Newton’s gradient method.

The proposed algorithm is comprehensively summarized in Algorithm 1. In the algorithm process, it is noted that, unlike NDT based on probability distribution, the information of feature points can be used separately to improve registration performance. Yet the voxelized mapping process during the initialization part is the same as NDT; this may result in the loss of local statistical information on the map as an inherent drawback.
**Algorithm 1. Register scan points**$\chi^{scan}$**to map**$M^{map}$**using NDT-P2P**NDT-P2P $\left(\;\chi^{scan}∋\left\{\;x_{1},x_{2},\cdots ,x_{i}\;\right\},\;\;M^{map}∋\left\{\;m_{1},m_{2},\cdots ,m_{i}\;\right\}\;\right)$
  1:   { Initialization : } same as NDT in Reference [11]
   2:   { Points Extraction : }
   3:   Allocate feature group structure $\chi^{key}$
   4:   Extract feature points $x_{key}\;$that contains $\chi^{scan}$
   5:   Store feature points $x_{key}$ in feature group $\chi_{\left(i\right)}^{key}$
   6:   **if** feature group size > window size *j*
**do**
   7:      Remove a *j-*th prior feature point in feature group
   8:   **end if**
   9:   **for** all feature group $x_{key}\in \chi_{\left(k\right)}^{key}$
**do**
   10:      $\chi_{\left(i\right)}^{key}=\left\{x_{key}^{1},x_{key}^{2},\cdots ,x_{key}^{j}\right\}\leftarrow$ all feature points within *i*-th group
   11:      $\mu_{key}\leftarrow \frac{1}{j}\sum_{k=1}^{j}x_{key}^{k}$
   12:      $\Sigma_{key}\leftarrow \frac{1}{j}\sum_{k=1}^{j}\left(x_{key}^{k}-\mu_{key}\right){\left(x_{key}^{k}-\mu_{key}\right)}^{T}$
   13:   **end for**
   14:   { Registration : }
   15:   **While** not converged **do**

   16:      score←0
   17:      g←0
   18:      H←0
   19:      **for** all points $x_{i}\in \chi^{scan}$
**do**
   20:         **if**
$x_{i}$ is feature points **do**
   21:           find the closest point $x_{target}\in M^{map}$ from $x_{i}$
   22:         **else**
   23:           find the cell that contains $T\left(y,x_{i}\right)$
   24:         **end if**
   25:         *score* $\leftarrow score+p\left(T\left(y,x_{i}\right)\right)$ (see Equation (12))
   26:         update g
   27:         update H
   28:      **end for**
   29:      solve H Δy=−g
   30:      y←y+Δy
   31:   **end while**

### 3.3. INS Integration

**Figure 5:**
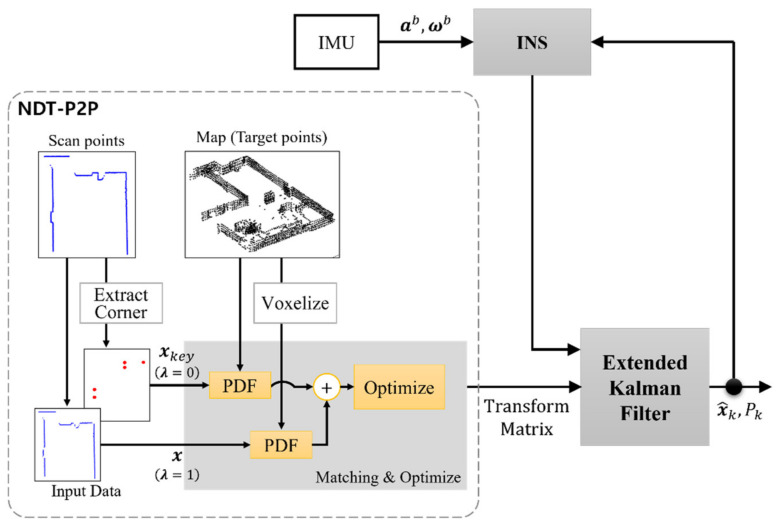
The block diagram of INS integration with NDT-P2P.

The registration process estimates the new pose information, which is slow because of 2D lidar’s update rate. For the integrated navigation, the registration output was combined with the INS mechanization, which implements a high update rate using the Extended Kalman Filter (EKF) framework. The output of proposed registration ‘NDT-P2P’ is a transform parameter that is further used as the measurement update of EKF. The conceptual block diagram of the presented method is illustrated in Figure 5.

First, a navigation state is defined as (14), and a simple error model considering a low-cost INS is described as (15).

(14) $$\delta x={\left[\;\begin{array}{ccccc} \delta p^{n} & \delta v^{n} & \delta \emptyset^{n} & \delta b_{a} & \delta b_{g} \end{array}\;\right]}_{15\times 1}^{T}$$

(15) $$\delta \dot{x}=F\;\delta x+G\;w$$

where

(16) $$F=\left[\begin{array}{ccccc} 0_{3\times 3} & I_{3\times 3} & 0_{3\times 3} & 0_{3\times 3} & 0_{3\times 3}\\ 0_{3\times 3} & 0_{3\times 3} & \left[\times C_{b}^{n}\left(a^{b}-b_{a}\right)\right] & -C_{b}^{n} & 0_{3\times 3}\\ 0_{3\times 3} & 0_{3\times 3} & 0_{3\times 3} & 0_{3\times 3} & -C_{b}^{n}\\ 0_{3\times 3} & 0_{3\times 3} & 0_{3\times 3} & 0_{3\times 3} & 0_{3\times 3}\\ 0_{3\times 3} & 0_{3\times 3} & 0_{3\times 3} & 0_{3\times 3} & 0_{3\times 3} \end{array}\right]$$

(17) $$G=\left[\;\;\begin{array}{cc} 0_{3\times 3} & 0_{3\times 3}\\ -C_{b}^{n} & 0_{3\times 3}\\ 0_{3\times 3} & -C_{b}^{n}\\ 0_{3\times 3} & 0_{3\times 3}\\ 0_{3\times 3} & 0_{3\times 3} \end{array}\;\;\right]$$

(18) $$w={\left[\;\begin{array}{cc} w_{acc} & w_{gyro} \end{array}\;\right]}^{T}$$

The superscript ‘*n*’ means the navigation frame, and the symbol ‘δ’ means an error state, i.e., true state minus estimated state. $p^{n}$, $v^{n}$, $\emptyset^{n}$ are position, velocity and attitude in the navigation frame, respectively. Especially, attitude error $\delta \emptyset^{n}$ means an angle vector for the 3-axis and is assumed to be small. $C_{b}^{n}$ is the Direction Cosine Matrix (DCM) that rotates from body frame to navigation frame. $b_{a}$, $b_{g}$ respectively represent an IMU sensor bias of accelerometer and gyroscope in the body frame. $\left(a^{b}-b_{a}\right)$ is the specific force vector of the accelerometer, and w represents the noise terms of the accelerometer and gyroscope.

The following is the measurement model of EKF. After registration, rotation matrix and corrected position are used in this updated process.

(19) $$\delta z_{k}=\left[\;\begin{array}{c} T\left(y,{\hat{p}}_{INS}^{-}\right)-{\hat{p}}_{INS}^{-}\\ \phi_{NDT} \end{array}\;\right]=H\;\delta x+\upsilon$$

(20) $$H=\left[\;\begin{array}{ccccc} I_{3\times 3} & 0_{3\times 3} & 0_{3\times 3} & 0_{3\times 3} & 0_{3\times 3}\\ 0_{3\times 3} & 0_{3\times 3} & I_{3\times 3} & 0_{3\times 3} & 0_{3\times 3} \end{array}\;\right]$$

(21) $$R=E\left\{\;\upsilon \upsilon^{T}\;\right\}=\left[\;\begin{array}{cc} R_{p} & 0_{3\times 3}\\ 0_{3\times 3} & R_{\emptyset } \end{array}\;\right]$$

where $T\left(y,{\hat{p}}_{INS}^{-}\right)$ denotes the corrected position, obtained by transforming estimated position ${\hat{p}}_{INS}^{-}$ by y. $\phi_{NDT}={\left[\phi_{N},\phi_{E},\phi_{D}\right]}^{T}$ is the rotation part of transform parameter y in the navigation frame. The rotation of the transform parameter can be applied directly because the scan points measured by lidar in the body frame were converted to navigation frame, and registration was performed. H is the observation matrix, and R is the measurement noise. Measurement noise can be adjusted by considering lidar’s noise and INS error. Table 1 summarizes the employed EKF.

## 4. Simulation and Experiment

To verify the proposed algorithm, we conducted a simulation study as well as a test using a moving cart in an indoor corridor. We compared the results of NDT-P2P to other reference algorithms [11,23] and analyzed overall performance.

### 4.1. Simulation

A simulation study was carried out using a Unity-based flight simulator [26]. In this, a pre-built map consisting of 3D point cloud data is used and algorithm is verified through the virtual sensor data including accelerometer, gyroscope and point cloud. The simulation scenario assumes a drone to inspect the power transmission tower, where circular trajectory is generated, with heading oriented to the center of tower. Considering the effect of electromagnetic fields (EMF) from the high voltage power line, we assumed a GNSS denied flight environment.

**Figure 6:**
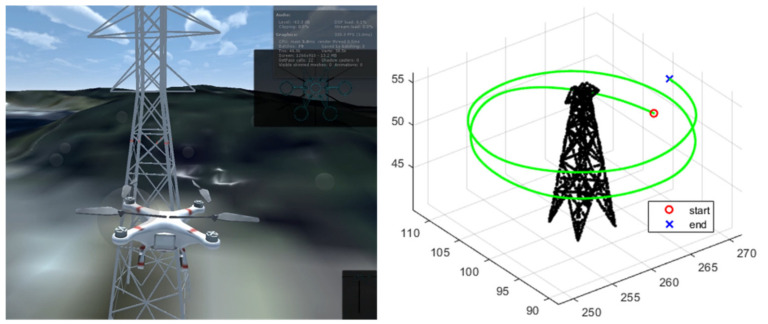
Unity-based simulator (**Left**) and flight path of simulation (**Right**).

Figure 6 shows the virtual environment implemented in the flight simulator. The right subplot shows a point cloud map of the transmission tower and the trajectory generated from the simulator. The total number of map points is 2050, which work as target points for the proposed algorithm. The measurement update rate of IMU and lidar is 100 Hz and 5Hz, respectively. The proposed algorithm was coded with C++ language including point cloud library (PCL) [27].

**Figure 7:**
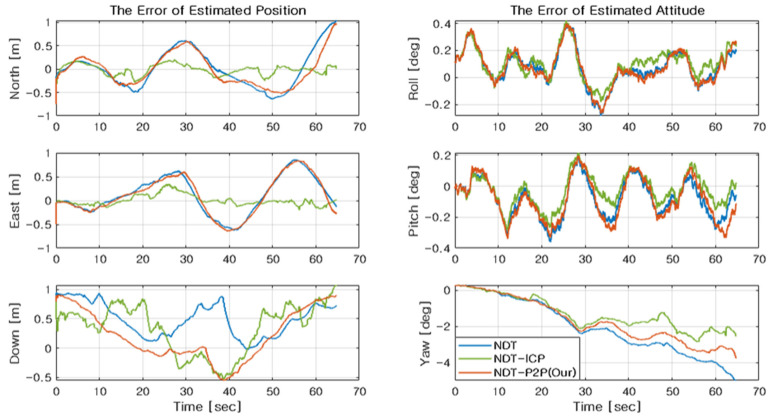
The errors of the estimated position and attitude.

As a result of the simulation, the errors of estimated position and attitude are plotted in Figure 7 and the resulting RMSE is summarized in Table 2. For performance comparison with the previous works, the conventional NDT and NDT-ICP is implemented and results are analyzed together. In performance analysis, the mean of the squared distance from the transformed point to the target can be used as an indicator of the accuracy of the transformation (TF accuracy). In Table 2, the TF accuracy of the methods with a point-to-point scheme shows better performance in general. However, due to 2D lidar, the TF accuracy cannot be regarded as a direct criterion for localization accuracy. For example, when a 3D cube map and 2D rectangular shaped points are assumed, registration is possible, but it may show uncertainty while estimating the pose. Therefore, we present localization results combined with INS using the transform parameter as the filter’s measurements.

In Figure 7, the blue line denotes the NDT algorithm, the green line denotes the NDT-ICP algorithm, and the red line denotes the proposed algorithm. It is observed that the proposed NDT-P2P typically demonstrates a position accuracy with sub-meter level throughout the simulation periods. Relatively, position accuracy is slightly degraded than NDT-ICP, yet a better result is obtained compared with a conventional NDT algorithm. Similar results are observed in the case of attitude estimation. The attitude estimation in the horizontal axis shows fairly good performance, in which the accuracy is obtained by sub-degree level. Without vertical measurement, the yaw estimation error is relatively enlarged, up to several degrees. This is because only point cloud data from the drone’s horizontal plane is acquired, considering a lightweight 2D lidar deployment condition. Therefore, the altitude is more vulnerable to sensor noise characteristics.

**Figure 8:**
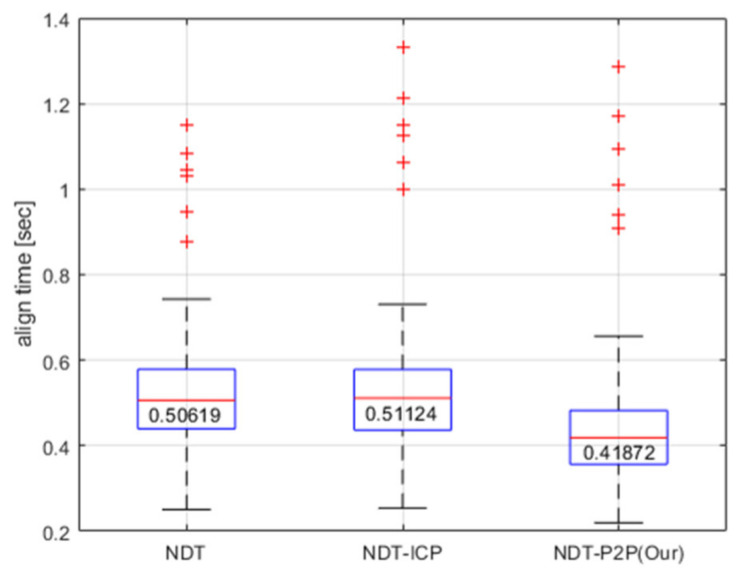
The process time by algorithm.

Next, computational efficiency is analyzed for each method. In the simulation result, it is observed that the registration algorithm’s process time takes the longest for NDT-ICP and is fastest for NDT-P2P. Figure 8 summarizes this result, where medians are displayed for easy notice. NDT-P2P searches for the closest point to feature point results more efficiently than NDT, which finds surrounding cells correspondingly. NDT-ICP takes a longer time than other algorithms because NDT-ICP performs NDT and then ICP sequentially. Although the proposed NDT-P2P includes feature point extraction time, total computational efficiency is achieved with the suggested scan point classification strategy, while estimation accuracy is maintained with a competent level of performance.

### 4.2. Experiment

**Figure 9:**
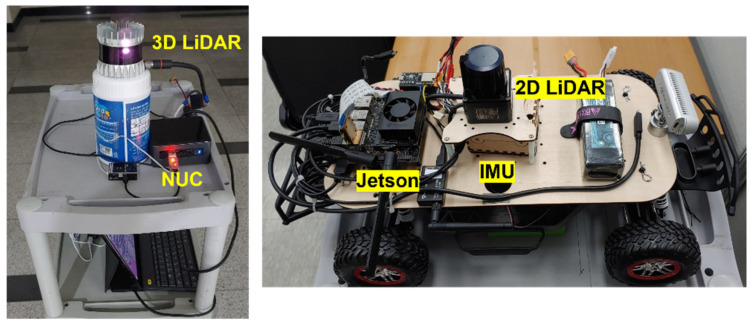
Test equipment.

Unlike SLAM that performs mapping and localization simultaneously, this study aims mainly to analyze a vehicle’s localization performance with the proposed algorithm in a situation where a 3D point cloud map exists. As shown in Figure 9, the measurement system is configured on a moving cart. Then, a practical test is conducted around an indoor hallway environment. Consistent with the simulation study, limited scan points in the horizontal plane are efficiently used for localization. The equipment used in the test is summarized in Table 3.

**Figure 10:**
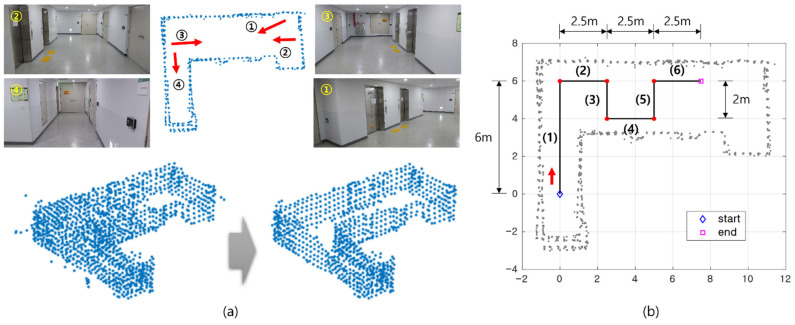
**(a**) Experimental environment and constructed indoor map. (**b**) Vehicle trajectory composed from line segment (1) to (6) shown in the 2D map.

As an infrastructure, a surrounding map is constructed using 3D lidar measurement and mapping algorithm. As shown in the left subplot of Figure 9, the system was configured with 3D lidar and a points cloud map about the hallway was constructed. A representative lidar slam algorithm, the LeGO-LOAM [28], is used for the mapping purpose. Figure 10a illustrates an indoor environment and the point cloud map created. As a result of mapping, points on the ceiling and floor side are observed. In applying the NDT-P2P algorithm, these points on the ceiling and floor are practically removed from the reference map as shown in the right lower plot in Figure 10a. In this process, since the map is assumed to be prebuilt, useless points such as outlier, ceiling and floor on the map were removed manually.

**Figure 11:**
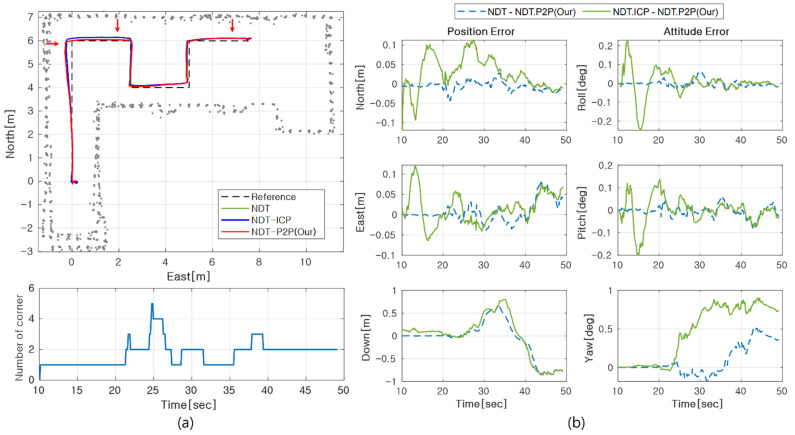
(**a**) Estimated position by algorithms and number of feature points extracted. (**b**) The error of position and attitude by NDT, NDT-ICP based on NDT-P2P.

Next, in localization, experiments were conducted using 2D lidar and IMU. The update rate of 2D lidar and IMU is 10 Hz and 100 Hz, respectively. In accordance with lidar’s update rate, the proposed NDT-P2P is updated in every 10 Hz. Figure 10b shows a test trajectory, where the heading of the moving platform always orients to the moving direction. The navigation results through this trajectory test are shown in Figure 11a. In addition, the number of feature points extracted for each epoch is shown. Unlike simulations that use virtual sensor data, the experiment used down-sampled points. This point cloud processing can reduce noise and enable robust corner extraction.

In Figure 11a, the upper subplot shows the estimated position of each algorithm. In the figure, it can be observed that each method demonstrates a similar position estimate result. For a detailed analysis, estimation difference between methods is first shown in Figure 11b. In this plot, the blue dotted line denotes the error between NDT and NDT-P2P and the green line denotes the error between NDT-ICP and NDT-P2P. It is stationary in the period between 0 to 18 s, and the cart begins to move at approximately 18 s. The estimation result reveals that NDT-P2P yields a more similar performance to NDT than NDT-ICP. Based on the NDT-P2P result, there is a difference of 7 cm, 0.5 deg from NDT for position and attitude, respectively. There is also a difference of 12 cm in the position and 0.9 deg of attitude from NDT-ICP. It can be confirmed that the altitude error is relatively large compared to the horizontal position error due to the constrained measurement characteristics from 2D lidar.

**Figure 12:**
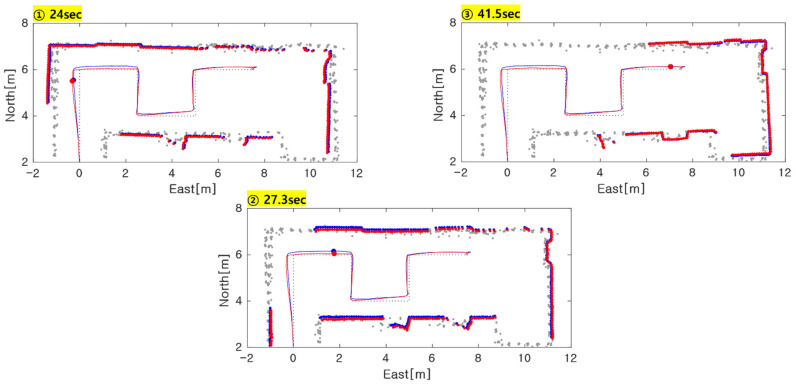
The point cloud data from estimated position by NDT-ICP, NDT-P2P.

In the previous performance analysis, time synchronous error characteristics from true path was unavailable since no infrastructure for reference trajectory is assumed. To resolve this, we introduced a traceback method that analyzes the difference between reference map and practical point cloud at particular time instants. When displaying point cloud data from the estimated position, the estimated accuracy can be predicted by the statistics of misalignment residuals between map and scan points. In particular, we focused on three locations for a quantitative analysis, which is marked with red arrows in Figure 11a. Each point corresponds to 24 s, 27.3 s, and 41.5 s. The first two instants represent the largest distance from reference path and the most significant differences between the proposed algorithm and NDT-ICP, respectively. Figure 12 shows the traceback results for two instants. The blue dot represents the estimated position and point cloud data of NDT-ICP, whereas the red dot is for NDT-P2P. At a 24 s point, it is observed that position of all algorithms is away from the reference path in the negative east direction. This means that the localization error was shown in the easterly direction, although the cart actually moved along the reference path. At a 27.3 s point, NDT-P2P is matched more closely to the map in the northward direction than NDT-ICP. In this instant, it can be concluded the proposed NDT-P2P method is more accurate than NDT-ICP. At a 41.5 s point, little difference is observed between NDT-ICP and NDT-P2P.

A further quantitative error analysis is performed based on the structure of the reference path during partial intervals. For example, the eastern error from the reference path can be obtained assuming a negligible deviation to the east direction when the cart is moving in the northward direction. On the contrary, the north error from reference path can be obtained when the cart is moving to the east direction. Table 4 shows the RMS error of fractional interval according to each partial path as shown in Figure 10b. Except for periods in which the path changes, such as the turning head of the cart, only the section that moves straight with a fixed heading is considered. The NDT-ICP algorithm has the largest error during path-(2), which corresponds to the result in the middle subplot of Figure 12 (i.e., scan instant of 27.3 s). For path-(6), all three methods present similar error performance as indicated in the last subplot of Figure 12. In summary, the average performance of the proposed algorithm presents superior accuracy over other methods.

**Figure 13:**
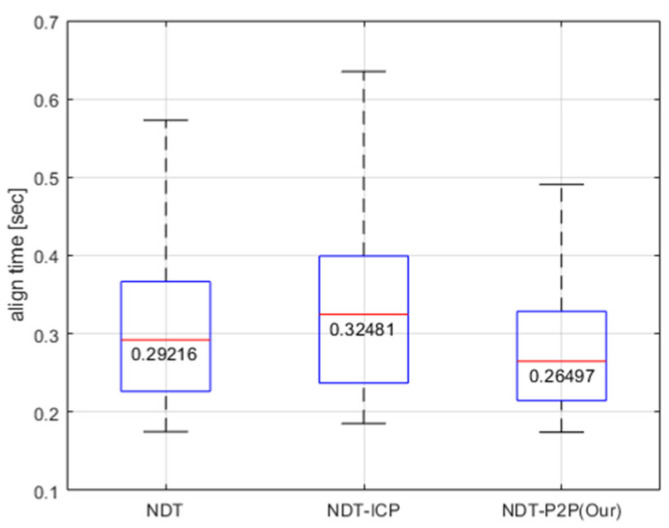
The process time by algorithm in an experiment.

In addition, Figure 13 illustrates the measurement update time for each registration algorithm. It shows the same tendency as Figure 8 of the simulation. Note that process time is mainly affected by the size of considered point cloud sets and the registration’s tunning parameters such as iteration, grid size, step size, etc. Thus, the difference in time between simulation and experiments is influenced by these tuning parameters.

## 5. Conclusions

NDT, as a point-to-normal distribution matching technique, has an advantage in process time and robustness over point-to-point matching, yet possesses defects in terms of aligning accuracy. In this study, we combine a matching technique between points on the basis of NDT using a statistical method. Specifically, the feature points (e.g., corner) are extracted from the point cloud data and applied in the cost function. In this way, the extracted corner points are accumulated during a window to generate probability distribution, which is further used in formulating the scan point optimization process.

The proposed algorithm is validated through simulation and experiment. We presented the localization performance of the vehicle via INS integration. In comparison with other methods, the proposed NDT-P2P has superior performance to NDT, which is widely used in applications, in aspects of accuracy and registration time. In an embedded system with limited resources, the computational efficiency of the algorithm is also an important factor. NDT-P2P can be a better choice than NDT-ICP in terms of accuracy. In summary, since traditional NDT is currently being used for mobile robots and autonomous vehicles, we expect that the proposed algorithm, inherited with its characteristics and benefits, can replace NDT. Furthermore, the presented algorithm can be more effectively deployed in the environment where feature extraction is facilitated, such as indoor, urban building areas, or environments with enlarged map complexity. In the future, it will be possible to analyze the performance according to the feature-to-scan points ratio. Feature points limited to corners can also be extended to lines, shapes, etc.

## Figures and Tables

**Table 1:** Extended Kalman Filter’s formula.

(Predict)	(Update)
$P_{k}^{-}=A\;P_{k-1}\;A^{T}+B\;Q\;B^{T}$	$K_{k}=P_{k}^{-}\;H^{T}\;{\left(H\;P_{k}^{-}\;H^{T}+R\right)}^{-1}$
$\left(A=I+F\;dt,\;B=G\;dt\right)$	$P_{k}=\left(I-K_{k}\;H\right)\;P_{k}^{-}$
${\hat{x}}_{k}^{-}=f\left({\hat{x}}_{k-1},u\right)$	${\hat{x}}_{k}={\hat{x}}_{k}^{-}+K_{k}\;\delta z_{k}$

**Table 2:** Transformation accuracy and RMSE for Figure 7.

		NDT Ref. [11]	NDT-ICP Ref. [23]	NDT-P2P (Proposed)
TF accuracy (m)		0.0438	0.0094	0.0387
Position (m)	North	0.401	0.112	0.344
	East	0.403	0.109	0.408
	Down	0.567	0.531	0.474
	2D	0.569	0.157	0.534
	3D	0.803	0.554	0.714
Attitude (°)	Roll	0.151	0.155	0.151
	Pitch	0.145	0.105	0.159
	Yaw	2.508	1.571	2.080

**Table 3:** The equipment used in the test.

	Mapping (Preprocess)	Localization
LiDAR	Ouster OS1-16 (3D LiDAR)	Hokuyo UST-20LX (2D LiDAR)
Main Board	Intel NUC (i7-8559U)	NVIDIA Jetson Xavier NX
IMU	-	ADIS16448

**Table 4:** RMSE for Figure 11a.

Path ID	NDT Ref. [11]	NDT-ICP Ref. [23]	NDT-P2P (Proposed)
Path (1)	0.1461	0.1189	0.1474
Path (2)	0.0411	0.1311	0.0329
Path (3)	0.0299	0.0175	0.0372
Path (4)	0.1090	0.1232	0.0993
Path (5)	0.1235	0.0828	0.1009
Path (6)	0.0858	0.0899	0.0872
Total	0.0535	0.5634	0.5048

## Data Availability

Not applicable.
